# Prevalence and risk factors of seafood-borne *Vibrio vulnificus* in Asia: a systematic review with meta-analysis and meta-regression

**DOI:** 10.3389/fmicb.2024.1363560

**Published:** 2024-03-06

**Authors:** Maryum Tanveer, Eurade Ntakiyisumba, Gayeon Won

**Affiliations:** College of Veterinary Medicine and Bio-Safety Research Institute, Jeonbuk National University, Iksan, Republic of Korea

**Keywords:** *Vibrio vulnificus*, meta-analysis, prevalence, meta-regression, seafood-borne disease

## Abstract

*Vibrio vulnificus* is a free-living marine bacterium associated with the contamination of fish and shellfish—the most consumed seafood in Asia. Owing to its potentially lethal clinical consequences, the consumption of seafood contaminated with *V. vulnificus* has become a growing public health concern. This systematic review with meta-analysis and meta-regression aimed to integrate data on the prevalence of seafood-borne *V. vulnificus* specifically in Asia and assess the potential risk factors that can influence the outcomes. A comprehensive literature search of four electronic databases yielded 279 relevant studies, among which 38 fulfilled the inclusion criteria. These selected studies were subjected to risk-of-bias assessment and data extraction by three independent researchers. A meta-analysis of the eligible studies estimated the overall prevalence of seafood-borne *V. vulnificus* in Asia to be 10.47% [95% confidence interval (CI): 6.8–15.8%], with bivalve shellfish, such as oysters, mussels, clams, and cockles being the most contaminated seafood. The highest prevalence was reported in Japan, where 47.6% of the seafood samples tested positive for *V. vulnificus*. The subgroup and meta-regression analyses identified three potential covariates—detection method, publication year, and country—associated with between-study heterogeneity. Furthermore, data visualization displayed the variations in *V. vulnificus* prevalence across the studies, associated with differences in sample type, sample size, and sampling stage. This study provides valuable insights into the prevalence of *V. vulnificus* in fish and shellfish across the entire Asian continent and highlights the potential factors that cause variation in the prevalence rates among the studies. These findings underscore the importance of enhancing hygiene measures throughout the seafood supply chain to mitigate *V. vulnificus* infection risks and ensure the safety of consumers.

## 1 Introduction

*Vibrio vulnificus* is a gram-negative opportunistic pathogen that is naturally found in marine environments where water temperature ranges from 9 to 31°C (Strom and Paranjpye, 2000; Heng et al., 2017). It is a highly lethal seafood-borne pathogen with a case-fatality rate greater than 30% (Lydon et al., 2021). A wide range of virulence characteristics, such as the ability to acquire iron, cytotoxicity, high motility, presence of a lipopolysaccharide capsule, and expression of adhesive proteins, allows this pathogen to overcome the immune responses initiated by infected individuals (Jones and Oliver, 2009). Generally, the concentration of *V. vulnificus* is quite low in estuarine waters and suspended microscopic organisms (<10 CFU/mL). However, when shellfish filter-feed on zooplankton, phytoplankton, and the decaying organic matter or detritus present in the water, the pathogen multiplies exponentially within their gut and tissues, reaching a level of 10^5^ CFU/g (Strom and Paranjpye, 2000; Oliver, 2015). This pathogen has also been reported in the epidermis, gills, and digestive tracts of fish (Strom and Paranjpye, 2000; Adebayo-Tayo et al., 2011). The primary infection caused by the ingestion of contaminated raw or undercooked seafood results in gastroenteritis. In extreme cases, the bacterium can enter the bloodstream, leading to a highly lethal systemic infection known as septicemia, which results in an average mortality rate of more than 50% (Jones and Oliver, 2009). Infection can also occur through the exposure of open wounds to contaminated seawater or seafood, which can lead to wound infections such as necrotizing fasciitis and cellulitis (Coerdt and Khachemoune, 2021). Depending on the severity of the infection, necrotizing fasciitis treatment may involve surgical interventions, such as fasciotomy or even amputation, with an associated mortality rate of approximately 15% (Bross et al., 2007). The growth and pathogenicity of this bacterium are highly correlated with iron availability in the body fluids of the host organism (Miyamoto et al., 2021); hence, immunocompromised individuals with pre-existing liver disease, chronic renal failure, diabetes mellitus, or thalassemia are more prone to *V. vulnificus* infections and are at high risk of fatality, as these conditions are associated with an excess of iron in the body (Chung et al., 2006; Liu et al., 2006). Although the infected patients generally undergo antimicrobial treatment after the onset of clinical symptoms, the emergence of antimicrobial resistance in *V. vulnificus* isolates has made empirical therapy more challenging (Liu et al., 2006). Despite the low incidence of *V. vulnificus* infection, its severe disease potential and high fatality rate make it imperative to investigate its prevalence and associated risk factors to develop preventive measures for the safety of seafood consumers.

Asian countries consume seafood at a higher rate than the global average because of their coastal locations and abundant seafood supply, making them more susceptible to *V. vulnificus* infections (Forman et al., 2008; Wai et al., 2021). The warming of marine waters owing to global climate change has also contributed to the proliferation of this pathogen across Asia (Baker-Austin et al., 2010). Several cross-sectional studies conducted in Asia have reported the prevalence of *V. vulnificus* in seafood, which has shown the potential risk of this pathogen in the region. However, no attempt has been made to integrate these studies to estimate the pooled prevalence of *V. vulnificus* quantitatively and comprehensively, despite its severe clinical consequences. To the best of our knowledge, the present study provides the first systematic review and meta-analysis to investigate the overall prevalence of *V. vulnificus* in seafood reservoirs of the entire Asian continent. The overarching goal was to compile scientific studies, rigorously selected by *a priori*-determined criteria, to generate a more accurate effect size (Crowther et al., 2010; Field and Gillett, 2010). Subgroup and meta-regression analyses were conducted to explore the potential variables underlying the heterogeneity among the selected studies (Thompson and Higgins, 2002). These findings are of public health significance as they aid in appraising intervention strategies and determining future research priorities to reduce human vulnerability to seafood-borne *V. vulnificus* infections.

## 2 Materials and methods

### 2.1 Study design

To determine the prevalence and risk factors of seafood-borne *V. vulnificus* in Asia, a systematic review and meta-analysis were conducted following the “Preferred Reporting Items for Systematic Reviews and Meta-Analyses Protocol” (PRISMA-P) guidelines (Page et al., 2021) (Supplementary Table S1). The research question was formulated in the “population, exposure, comparator, and outcome” (PECO) format, wherein “population” indicated seafood samples and “exposure” referred to *V. vulnificus*. Since this systematic review and meta-analysis focused on prevalence, the term “comparator” was not applicable, hence omitted. The presence or absence of *V. vulnificus* in seafood samples obtained at any stage of the seafood supply chain was considered the “outcome” of each primary study.

### 2.2 Search strategy

A comprehensive literature search of four electronic databases (PubMed, Web of Science, Scopus, and Cochrane Library) was performed to identify relevant studies reporting the prevalence of *V. vulnificus* in seafood, published until December 31, 2022. For this purpose, an appropriate search string was developed using a combination of free text and controlled vocabulary terms such as the name of the concerned pathogen and its various pseudonyms, types of common seafood and their habitats, the names of all Asian countries, and prevalence-related keywords. The search terms were combined with Boolean operators (AND/OR), an asterisk (*), and parentheses in a meticulously selected manner to ensure the retrieval of relevant information (a detailed search string is provided in Table 1). Language restrictions were not imposed on the search, which led to the discovery of non-English articles published in Chinese, Japanese, and Korean that were translated and included in this study. All references were managed with the Endnote citation manager (Endnote X9, Clarivate, Philadelphia, PA, United States). After importing to Endnote, duplicate studies were removed. Additionally, a manual search of the reference lists in downloaded articles was conducted to identify any potential studies that might have been inadvertently overlooked during the initial search process.

**Table 1:** The eligibility criteria and the search string for assessing the prevalence of seafood-borne *Vibrio vulnificus* in the Asia countries.

Criteria	Inclusion	Exclusion
Study type	Cross-sectional studies	Cohort studies Case–control studies Ecological studies Non-primary research (reviews and meta-analysis)
Population	Bivalves, crustaceans, cephalopods, and fish	Marine algae and other marine animals
Exposure	*V. vulnificus*	Pathogens other than *V. vulnificus*
Outcomes	Prevalence (or proportion positive)	Outcomes other than prevalence (e.g., genomic analysis, antibiotic resistance)
Region	All subregions of Asia	Regions other than Asia
Search string		
(*Vibrio vulnificus* OR lactose-positive vibrio OR *Beneckea vulnifica* OR flesh-eating bacteria) AND (seafood OR seafood dishes OR fish food OR fish OR shrimp OR prawns OR shellfish OR oyster OR crustacean OR lobster OR mollusk OR mussel OR freshwater OR marine water OR saltwater OR estuaries OR ponds OR rivers OR coastal area) AND (Asia* OR China OR India OR Indonesia OR Pakistan OR Bangladesh OR Japan OR Philippines OR Vietnam OR Turkey OR Iran OR Thailand OR Myanmar OR South Korea OR Iraq OR Afghanistan OR Saudi Arab OR Uzbekistan OR Malaysia OR Yemen OR Nepal OR North Korea OR Sri Lanka OR Kazakhstan OR Syria OR Cambodia OR Jordan OR Azerbaijan OR United Arab Emirates OR Tajikistan OR Israel OR Laos OR Lebanon OR Kyrgyzstan OR Turkmenistan OR Singapore OR Oman OR Palestine OR Kuwait OR Georgia OR Mongolia OR Armenia OR Qatar OR Bahrain OR Timor-Leste OR Cyprus OR Bhutan OR Maldives OR Brunei) AND (incidence OR prevalence OR occurrence OR cases OR widespread OR presence OR commonness OR event OR proportion OR phenomena)		

### 2.3 Eligibility criteria and screening

After removing duplicates, the remaining articles were screened manually against predetermined inclusion and exclusion criteria (Table 1). Three independent researchers assessed the eligibility of the studies with any disagreements resolved through consensus or arbitration by an independent reviewer. An initial screening of titles and abstracts was performed, followed by a final screening of the full texts. After two-level screening, articles that fulfilled the inclusion criteria were considered eligible and selected for qualitative (systematic review) and quantitative (meta-analysis) synthesis. Non-accessible articles were obtained from the Foreign Research Information Center of Jeonbuk National University^1^.

### 2.4 Risk-of-bias assessment

To evaluate the internal and external validity of the included studies, the risk-of-bias (RoB) assessment was carried out by three independent researchers using the critical appraisal checklist of the Joanna Briggs Institute (JBI) for prevalence studies (Martin, 2017). The checklist contains nine RoB criteria related to the target population, sampling strategy, clarity in describing study subjects, reliability and validity of diagnostic methods, data collection method, response rate, likelihood of nonresponse bias, adequacy of sample size, and appropriateness of statistical analysis. Only seven of them were evaluated because two survey-type criteria (in terms of participants’ response rates) did not apply to this study. Based on seven of the nine RoB criteria included in the checklist, each study was rated as having either a low, high, or unclear RoB. A final decision was reached through consensus among the researchers, and disagreements were resolved through arbitration by an independent reviewer. The overall RoB results of the included studies were graphically presented using the Review Manager software RevMan 5.4.1.

### 2.5 Data extraction

After validating quality of the eligible studies through RoB assessment, relevant information was collected and organized in a Microsoft Excel spreadsheet to facilitate data analysis. The extracted data included several key variables, such as author name, publication year, country of study, sample type (fish, shellfish, or other types of seafood), sampling stage (harvest or post-harvest), prevalence rate of *V. vulnificus* (number of total and positive samples), and detection method (biochemical tests or PCR). In cases where more than one trial was conducted in a single study (e.g., using different sample types or sampling stages), the data were extracted separately from each trial.

### 2.6 Meta-analysis

Given the nature of this systematic review, which included studies from various Asian regions with potentially varying conditions and populations, a random-effects meta-analysis was employed to provide a more conservative estimate of the summary effect size while accounting for the expected heterogeneity. It considers both within-study and between-study variations, making it a suitable choice for pooling data from diverse sources (Harrer et al., 2021). In the random-effects model, the pooled prevalence estimate is calculated as the weighted average of the observed prevalence estimates from individual studies. The weight assigned to each study is determined by the inverse of its variance. Consequently, the studies with smaller variances (more precise estimates) receive greater weights, while the studies with larger variances (less precise estimates) receive smaller weights. This approach ensures that more reliable estimates contribute more substantially to the overall pooled estimate, reflecting the varying levels of precision across the included studies (Lipsey and Wilson, 2001). To fulfill the assumption of normal distribution and stabilize variances, the prevalence estimates from individual studies were subjected to a logit transformation (Barendregt et al., 2013). A Generalized Linear Mixed Model (GLMM) was then employed for pooling the transformed data, and between-study variance (*τ*^2^) was calculated by the Maximum Likelihood (ML) estimator (Lin and Chu, 2020). For ease of interpretation, the prevalence rates and the corresponding 95% confidence intervals (CIs) of the logit model were back-transformed to their original forms and represented as percentages. The between-study heterogeneity was assessed using the *Q* test and *I*^2^ statistics, which accounts for the percentage of the total variability in the effect estimates caused by true heterogeneity rather than random chance. Heterogeneity was considered significantly high if the *Q* test yielded a significant *p*-value (<0.05) and *I*^2^ was greater than 50% (Higgins and Thompson, 2002; Deeks et al., 2019). The findings of the meta-analysis were visually presented as forest plots. The methodology for calculating the logit, standard error, inverse variance weight for individual studies, and the weighted average proportion using logit transformation followed the approach outlined by Wang (2023). In order to assess the robustness of the meta-analysis findings, a sensitivity analysis using the leave-one-out method was also conducted. This approach aimed to identify any outliers or influential studies that could potentially impact the pooled prevalence estimate (Wang, 2023).

Given the anticipated heterogeneity in prevalence studies, it is commonly recommended to conduct moderator analyses to delve deeper into the reasons behind between-study heterogeneity. Similarly, in this meta-analysis, six *a priori*-determined covariates—publication year, country, detection method, sample size, sample type, and sampling stage—were presumed to be associated with the variations in the prevalence rate of *V. vulnificus* across the studies. Subsequently, comprehensive moderator analyses, employing subgroup and meta-regression analyses were conducted to investigate the potential factors underlying between-study heterogeneity using the above-mentioned variables. The statistical program “R” (*version 4.1.2*) and accompanying R-studio (*version 1.4.1106*) was utilized to perform all these analyses by employing “metafor” (*version 3.8–1*) and “meta” (*version 5.5–0*) packages.

### 2.7 Meta-regression and data visualization

To quantify the extent of observed variability in prevalence estimates across the studies, explained by the selected variables, meta-regression analyses were conducted using the R package “MuMIn” (*version 1.43.17*) and “dmetar” (*version 0.0.9000*). Categorical variables were encoded as dummy variables to allow their inclusion in the regression models. Initially, univariate meta-regression analyses were performed for each of the six covariates independently to assess their individual impact on the between-study heterogeneity. Covariates with a *p*-value ≤ 0.1 were then considered for inclusion in the multivariate meta-regression model, allowing for a simultaneous examination of their impact. To enhance the robustness of multivariate meta-regression model, the multi-model inference approach was employed. This approach systematically considers and compares multiple models, each with a different combination of covariates, rather than relying on a single “best” model. Model averaging was done by assigning weight to each model based on its performance (Harrer et al., 2021). The weights assigned to each model were determined by Akaike’s information criterion (AICc). The model with the lowest AICc was identified as the best optimal model, indicating a balance between goodness of fit and the number of predictors (Field and Gillett, 2010). Subsequently, the coefficients of this model were utilized to formulate the regression equation. A plot was generated to visually represent the averaged importance of predictors across all models. This involves ranking predictors according to their relative contribution in explaining the variability across prevalence estimates. For the variables that were not included in the multiple meta-regression model, a graphical representation was utilized to visualize their impact on the prevalence estimates across the studies using the R package “ggplot2” (*version 3.3.6*).

### 2.8 Publication bias

Publication bias refers to the selective publication of the studies based on their statistical significance, magnitude and direction of findings (Wang, 2017). It is a threat to systematic reviews and meta-analyses, as it could lead to a biased estimate of the effect size. In this study, the likelihood of publication bias on the prevalence of seafood-borne *Vibrio vulnificus* in Asia was evaluated through visual inspection of the contour-enhanced funnel plot symmetry (Palmer et al., 2008). To avoid subjective inferences and enhance interpretation, publication bias was quantitatively estimated using Egger’s regression test (Egger et al., 1997). Furthermore, a trim-and-fill method was employed to generate a non-biased effect size by imputing missing studies into the funnel plot (Palmer et al., 2008).

## 3 Results

### 3.1 Literature search and selection

**Figure 1:**
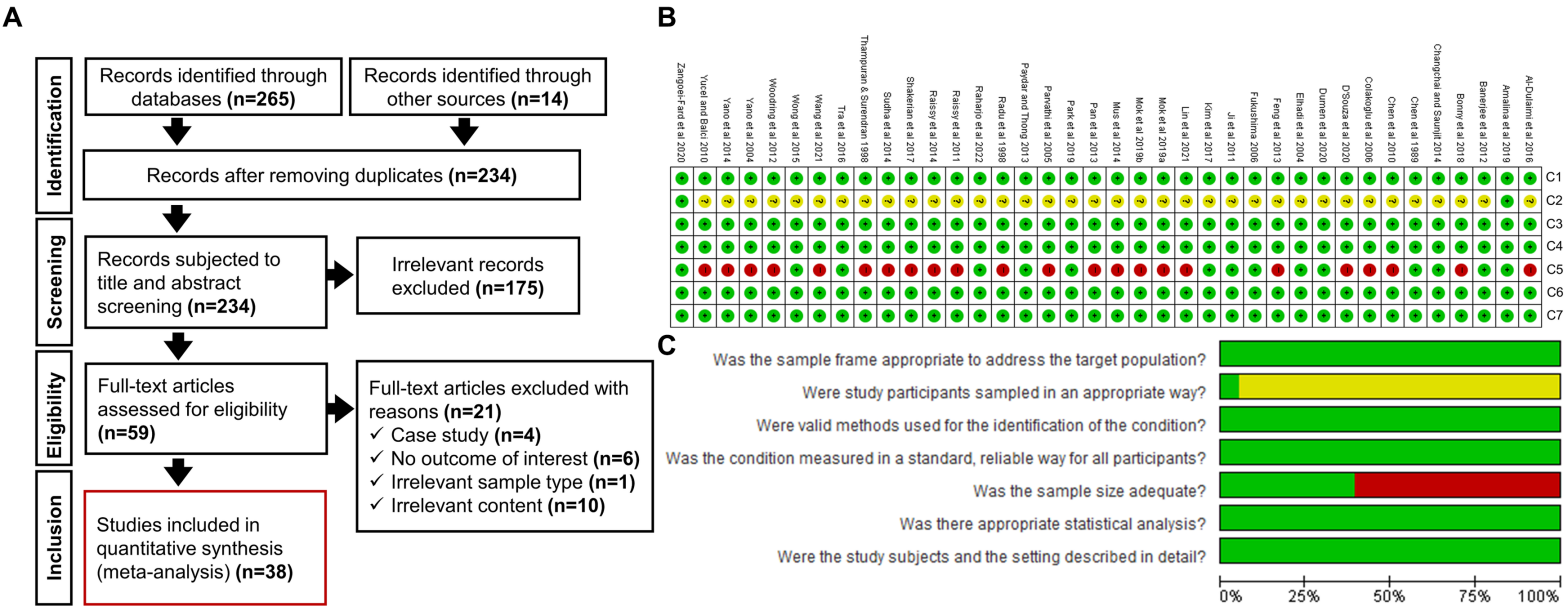
**(A)** Selection of studies used in the systematic review and meta-analysis (PRISMA flow chart) to determine the prevalence of seafood-borne *Vibrio vulnificus* in Asia. **(B)** Risk-of-bias summary presenting judgements about each RoB item across included studies (A green circle with a plus sign (+) denotes a low risk-of-bias, a red circle with a negative sign (−) denotes a high risk-of-bias, and a yellow circle with a question mark (?) indicates an unclear risk-of-bias). **(C)** Risk-of-bias graph presenting judgements about each RoB item as percentages across all included studies.

A systematic literature search following the PRISMA guidelines was conducted to ensure transparency in reporting our research findings. Figure 1A shows the number of research articles retrieved at each stage of the process. An initial literature search of electronic databases and other sources yielded 279 documents. After removing the duplicates, 234 records remained. A further 175 studies were eliminated after the preliminary title and abstract screening, and another 21 documents were removed because of irrelevant content found during the full-text screening. Finally, 38 articles were found to be eligible for inclusion in this meta-analysis.

### 3.2 RoB assessment

Before extracting data from eligible articles, RoB was assessed against the seven relevant quality criteria (C1–C7) mentioned in the JBI critical appraisal checklist to evaluate the internal and external validity of data (Martin, 2017). A descriptive justification for each judgment was presented, with figures showing the RoB assessment (Figures 1B,C). A score sheet for the quality evaluation of all included studies is also provided in Supplementary Table S2. Five of the seven examined criteria (C1, C3, C4, C6, and C7) indicated a low RoB for each study. The criteria for the appropriateness of the sample type (C1) ensured that the intended target population aligned with the inclusion requirements (Table 1). To authenticate the detection approaches, the criteria for evaluation (C3) guaranteed that each positive sample was identified efficiently, and all samples underwent the same methodology to detect the pathogen of interest (C4). Similarly, the criteria for measuring the outcome included a clear depiction of the number of positives, the number of samples tested, and/or the ability to calculate the prevalence (C6) for a specific subgroup (C7). In contrast, the RoB criteria for sampling strategy (C2) were unclear in 95% of the studies. This is primarily because random sampling was mentioned in only two studies (Amalina et al., 2019; Zangoei-Fard et al., 2020), leaving out 36 other studies. Finally, for the studies that did not report their sample size calculation, the adequacy of the used sample size was evaluated by using the formula suggested by Naing et al. (2006). By employing that approach, a minimum sample size of 150 was deemed appropriate for precise *V. vulnificus* prevalence estimation. Consequently, 23 of the 38 studies (61%) were judged to be at a high RoB on the adequate sample size criterion (C5) because they used less than 150 samples. Overall, all the studies fulfill at least five of the seven quality criteria, thereby warranted to be at low RoB.

### 3.3 Descriptive characteristics of eligible studies

**Figure 2:**
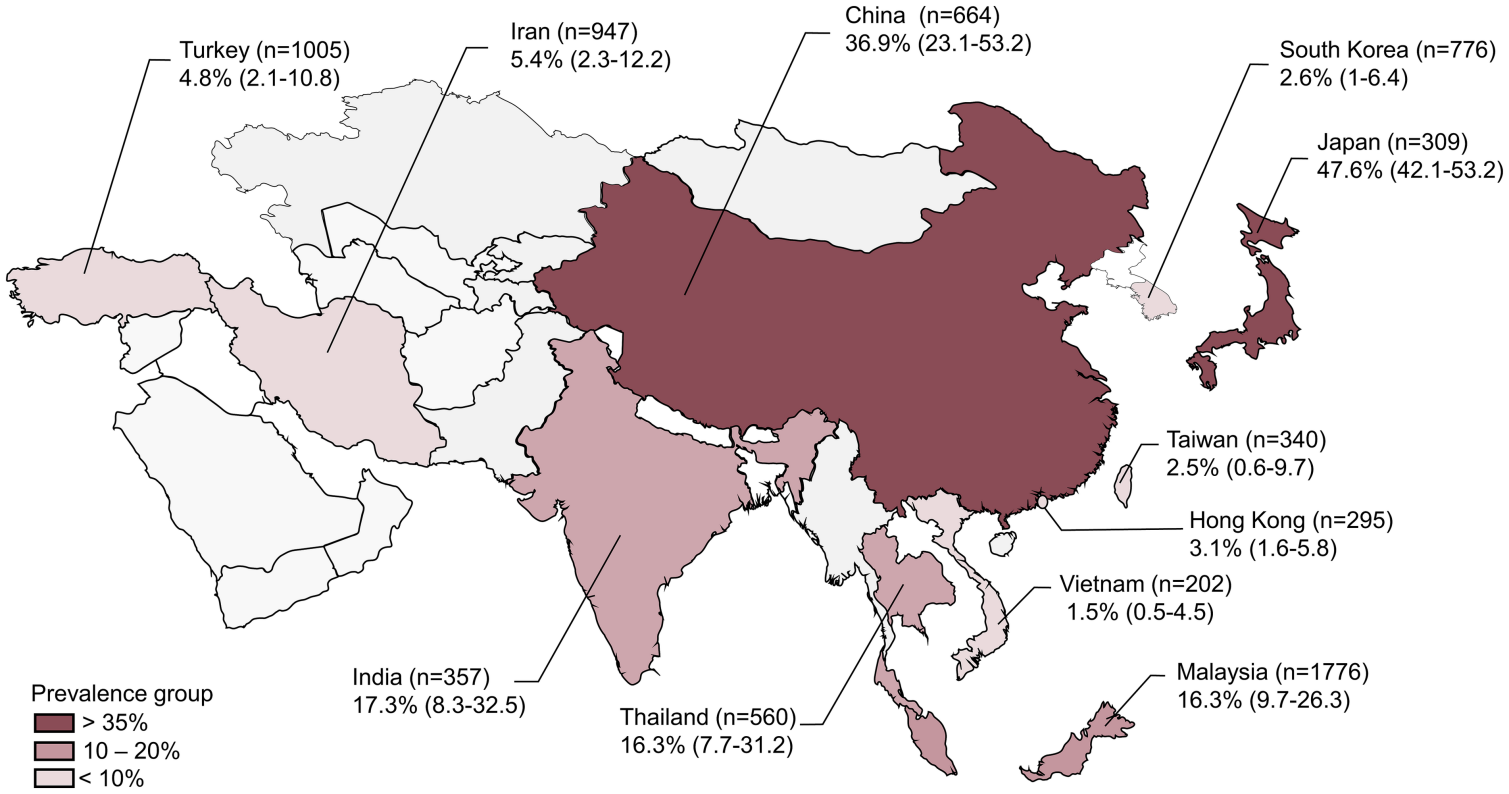
Prevalence of *Vibrio vulnificus* in the Asian countries included in the current meta-analysis with their 95% confidence intervals (*n* represents the number of samples reported from each country).

The 38 cross-sectional epidemiological studies with an explicit number of samples collected for the detection of *V. vulnificus* prevalence were included in this meta-analysis (Supplementary Table S3). These studies were conducted between 1989 and 2022 in 11 countries located in West Asia (Turkey and Iran), South Asia (India), East Asia (China, Japan, Hong Kong, South Korea, and Taiwan), and Southeast Asia (Thailand, Malaysia, and Vietnam). Figure 2 shows a map of the Asian countries included in the meta-analysis along with the reported sample size and prevalence. Among the eligible studies, the prevalence of *V. vulnificus* was investigated in bivalves (oysters, mussels, clams, cockles, and scallops), crustaceans (shrimps, prawns, crabs, crayfish, and lobsters), cephalopods (squids and octopuses), fish (salmon, tuna, carp, tilapia, and sea bass), and other seafood (sea snails and sea urchins). Various stages of the seafood supply chain were categorized into two primary groups: “harvest” and “post-harvest.” Each group represents the point in the supply chain from which samples were collected. The “harvest group” encompasses the studies where seafood samples were directly obtained from natural habitats such as oceans, seas, rivers, lakes, and other water bodies. On the other hand, the “post-harvest group” comprises the studies where seafood samples were collected from seafood markets, retail shops, or other seafood stores. Along the seafood supply chain, 12 studies collected samples at the harvest stage, 23 at the post-harvest stage, and 3 studies collected samples from both stages. For the detection of *V. vulnificus* in seafood samples, all the included studies initiate with sample pre-enrichment (primarily utilizing alkaline peptone water), followed by pathogen cultivation and isolation on selective agar media (predominantly TCBS agar). For the species identification, 25 of the 38 studies employed PCR assays (genotypic identification) while the remaining 13 studies relied only on biochemical tests (phenotypic identification) to confirm the presence of *V. vulnificus* (Supplementary Table S4).

### 3.4 Meta-analysis

**Figure 3:**
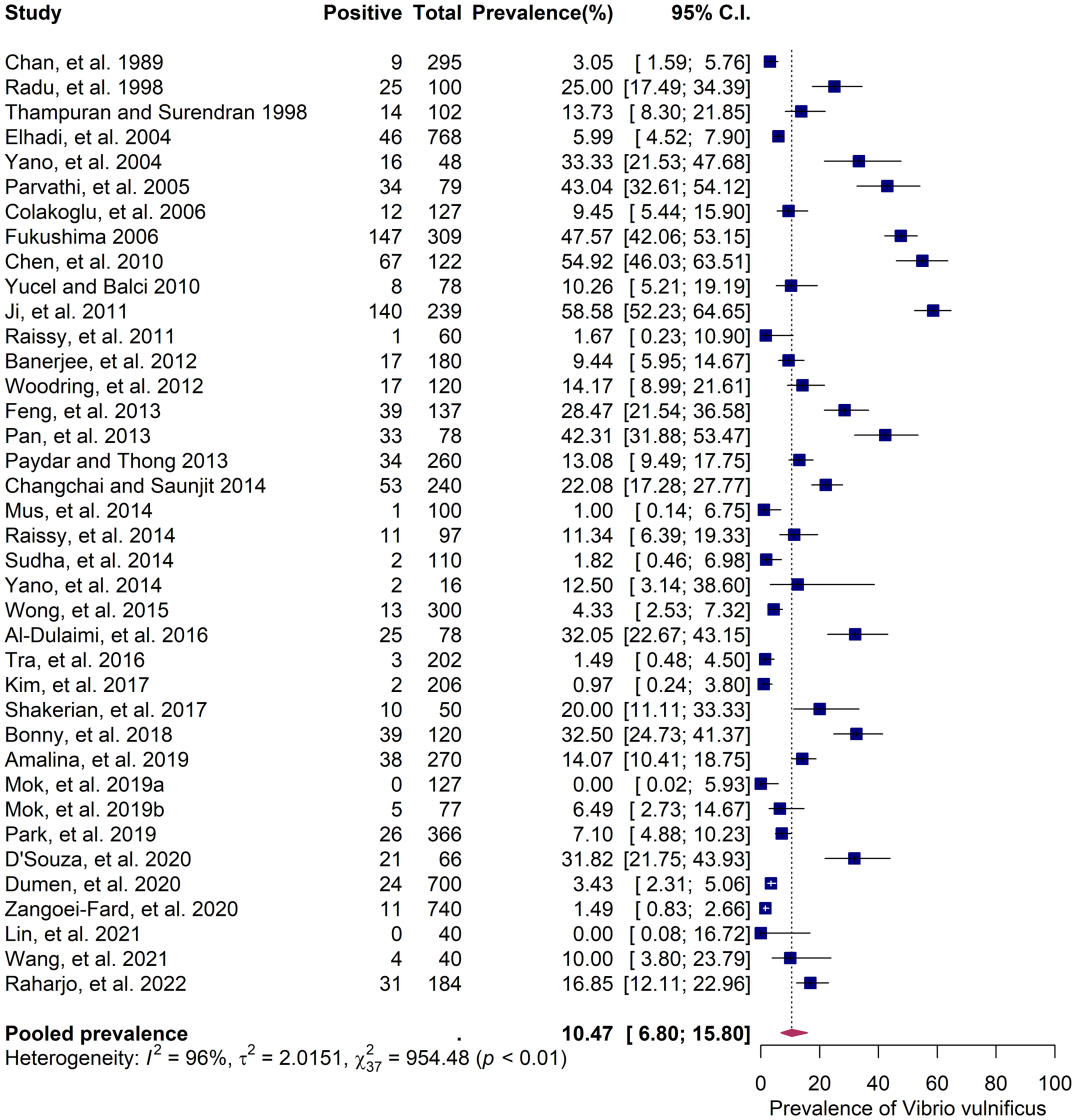
Forest plot showing the random-effects meta-analysis of 38 studies on the prevalence of seafood-borne *Vibrio vulnificus* in Asia. The blue squares represent the prevalence estimates of individual studies with their 95% confidence intervals (CIs), while the red diamond indicates the pooled prevalence estimate.

The 38 primary studies that reported the prevalence of *V. vulnificus* in seafood were evaluated using random effects meta-analysis. The estimated pooled prevalence was 10.47% (95% CI: 6.8–15.8), suggesting that approximately 7–16% of the seafood samples consumed in Asia contain *V. vulnificus* (Figure 3). *Q*-statistic indicated significant heterogeneity across the studies (*Q* = 954.48; *p*-value < 0.01; *I*^2^ = 96%). Despite the heterogeneous prevalence rates in the primary studies, the leave-one-out analysis demonstrated that no individual study substantially influenced the outcomes (Supplementary Figure S1). The leave-one-out prevalence estimates ranged from 11% (95%CI: 8–16) to 12% (95%CI: 8–18), suggesting that the results of the meta-analysis are robust and reliable. The Subgroup analyses based on six variables—country, publication year, detection method, sample type, sample size, and sampling stage—explore the potential sources of between-study heterogeneity among the prevalence estimates (Supplementary Table S5). The subgroup analyses revealed that country, publication year, and detection method were significantly associated with variations in the prevalence estimates (*p* < 0.05). Regarding countries, Japan had the highest prevalence with an estimate of 47.6% (95% CI: 42.1–53.2%), followed by China (36.9, 95% CI: 23.1–53.2%), India (17.3, 95% CI: 8.3–32.5%), Thailand (16.3, 95% CI: 7.7–31.2%), and Malaysia (16.3, 95% CI: 9.7–26.3%). A prevalence rate of less than 10% was reported in Hong Kong, South Korea, Taiwan, Vietnam, Turkey, and Iran (Figure 2). Regarding publication year, the studies published before 2015 showed a higher prevalence (15.02, 95% CI: 9.02–23.97%) than those published after 2015 (6.26, 95% CI: 3.23–11.82%). In terms of detection method, the studies that included the more sensitive PCR method for the detection of *V. vulnificus* exhibited a higher prevalence rate (14.28, 95% CI, 8.82–22.28%) compared to those that used conventional biochemical tests (5.72, 95% CI, 2.75–11.53%). For other covariates, including sample type, sample size, and sampling stage, the results showed that although variation was present among the subgroups, the difference was not statistically significant (*p* > 0.05). The quantitative impact of each covariate on the between-study heterogeneity of the estimated prevalence was further assessed using meta-regression.

### 3.5 Meta-regression and data visualization

**Figure 4:**
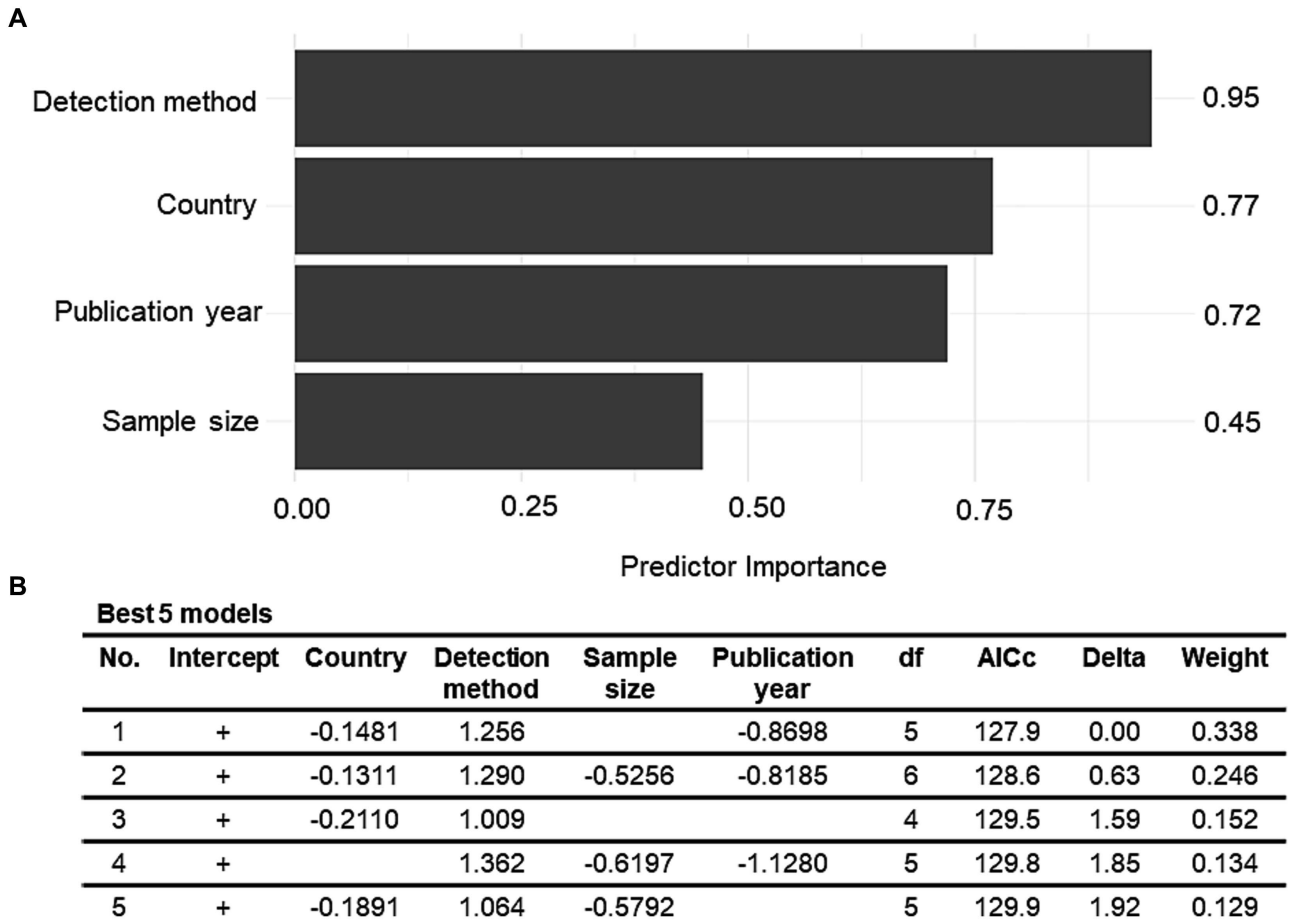
**(A)** Model-averaged predictor importance plot for a multiple meta-regression model of the *Vibrio vulnificus* prevalence data. **(B)** The best five multiple meta-regression models with the lowest value of corrected Akaike’s information criteria (AICc) for the *Vibrio vulnificus* prevalence outcome.

The relationship of between-study heterogeneity and variables of interest (country, publication year, detection method, sample type, sample size, and sampling stage) was examined using univariate and multivariate meta-regression analyses. The univariate meta-regression analysis revealed a significant relationship between country and between-study heterogeneity (*R*^2^ = 18.14%, *p* < 0.01). The findings suggest that the country accounted for 18% of the true heterogeneity in the observed prevalence rates across the studies. Similarly, the publication year and detection method were also significantly associated with between-study heterogeneity, with *R*^2^ values of 11.46 and 12.93%, respectively. Although sample size, sample type, and sampling stage contributed 9.48, 1.41, and 3.23%, respectively, to the true heterogeneity in the observed prevalence, their relationship was not significant (*p* > 0.05). The variables whose *p*-values were less than or equal to 0.1 in the univariable analyses were selected to construct a multivariable meta-regression model. Hence, the sampling stage and sample type were excluded because their *p*-values exceeded 0.1, whereas the other four variables were taken into account. The multivariate meta-regression model revealed that the combination of detection method, country, publication year, and sample size accounted for 48.16% of the heterogeneity across the prevalence estimates. Although this contribution was significant (*p* < 0.0001), the model did not entirely explain the reasons for the between-study heterogeneity. Owing to the presence of high residual heterogeneity (*I*^2^ = 93.19%, *p* < 0.0001), it is possible that additional factors that have not been considered, may contribute to the varying prevalence estimates. The predictor importance values revealed that the detection method had the highest contribution in the regression model (*r* = 0.95), followed by country (*r* = 0.77), publication year (*r* = 0.72), and sample size (*r* = 0.45) (Figure 4A). This implies that the reasons for the variability in *V. vulnificus* prevalence rates in Asia are multifactorial, with the aforementioned predictors being the most important. To minimize the risk of overfitting, a model with a small number of terms and the lowest value of AICc was considered most desirable (Figure 4B). Hence, the final model was:


$$\begin{array}{l} \mathrm{logit}\left(Y_{\mathrm{Prevalence}}\right)=-1.6881-0.1481\times \;\;X_{\mathrm{Country}}+1.256\\ \;\;\;\;\;\;\;\;\;\;\;\;\;\;\;\;\;\;\;\;\;\;\;\;\;\;\;\;\;\;\;\;\;\;\;\;\;\;\;\;\;\;\;\;\;\;\times X_{\mathrm{Detection method}}-0.8698\times X_{\mathrm{Publication year}} \end{array}$$

where logit (*Y*_Prevalence_) denotes the logit-transformed value of prevalence and *X*_Country_, *X*_Detection method_, and *X*_Publication year_ are the independent study variables.

**Figure 5:**
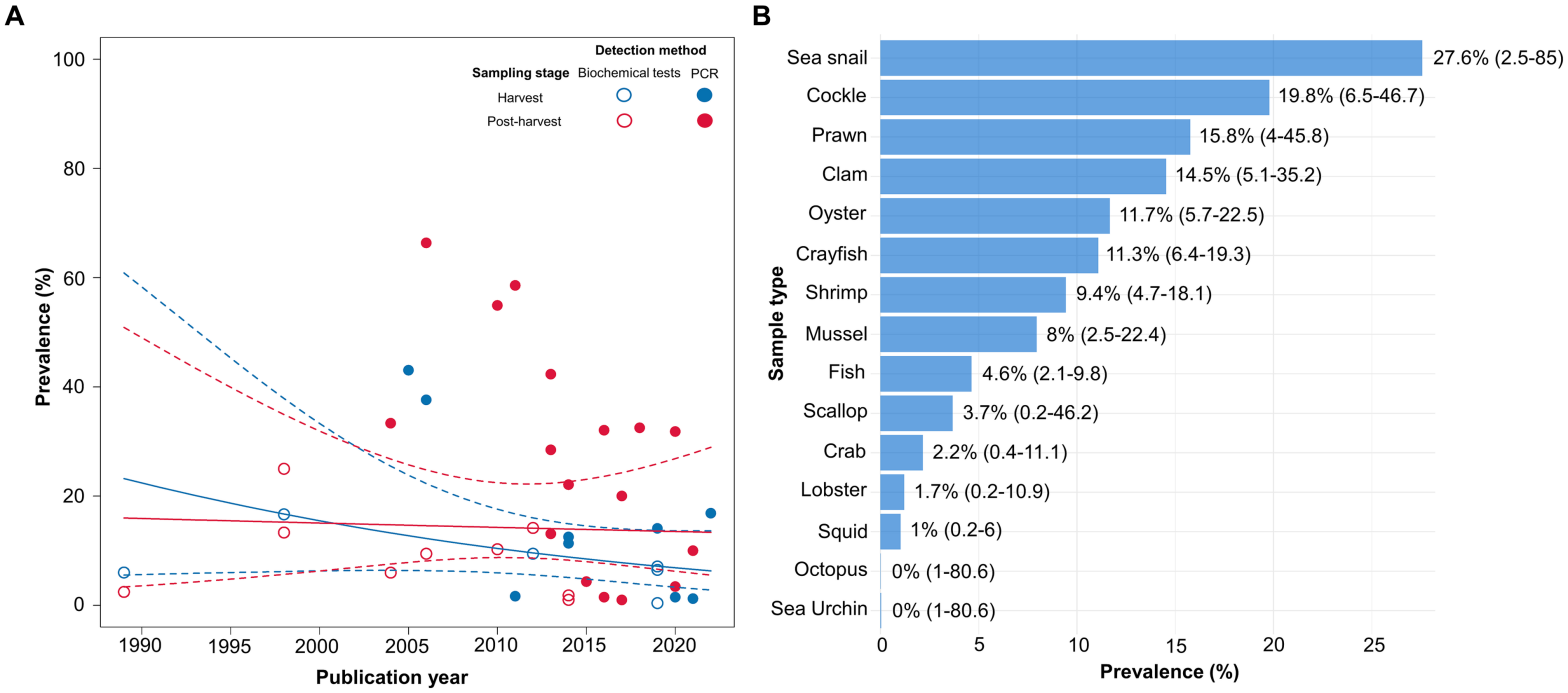
**(A)** Scatter plot showing the prevalence of *Vibrio vulnificus* over the years (1989–2022) regarding the sampling stage and the detection method. The solid line denotes the regression describing the association between the sampling stage and prevalence and the dashed lines indicate 95% confidence intervals (CIs) of the regression model. **(B)** The prevalence of *Vibrio vulnificus* in each sample type with the corresponding 95% CIs.

Finally, the data visualization was performed to examine the variations in the prevalence estimates across the studies based on the variables that were not considered in the multivariate meta-regression model. Given the importance of the detection method, it was also incorporated into the visualization graph to enhance the clarity of its impact on the prevalence rates among the studies. It was observed that the studies employing biochemical tests consistently reported prevalence rates below 25% over time, whereas those utilizing PCR showed a lower prevalence rate after 2015 (Figure 5A). In case of sampling stage, the subgroup analysis revealed that the prevalence of *V. vulnificus* in freshly harvested samples was 7.26% (CI: 3.5–14.47%), whereas a notably higher prevalence rate of 13.49% (95%CI: 8.2–21.41%) was observed in the samples collected at the post-harvest stage. Figure 5A shows that most post-harvested and all freshly harvested samples had lower prevalence rates after 2015. Regarding the sample type, the highest prevalence was detected in sea snails (27.6%), followed by cockles (19.8%), prawns (15.8%), clams (14.5%), oysters (11.7%), crayfish (11.3%), shrimps (9.4%), mussels (8%), and fish (4.6%). Conversely, *V. vulnificus* was not detected in octopuses or sea urchins, whereas scallops, crabs, lobsters, and squids had low detection rates (3.7, 2.2, 1.7, and 1%, respectively) (Figure 5B).

### 3.6 Publication bias

**Figure 6:**
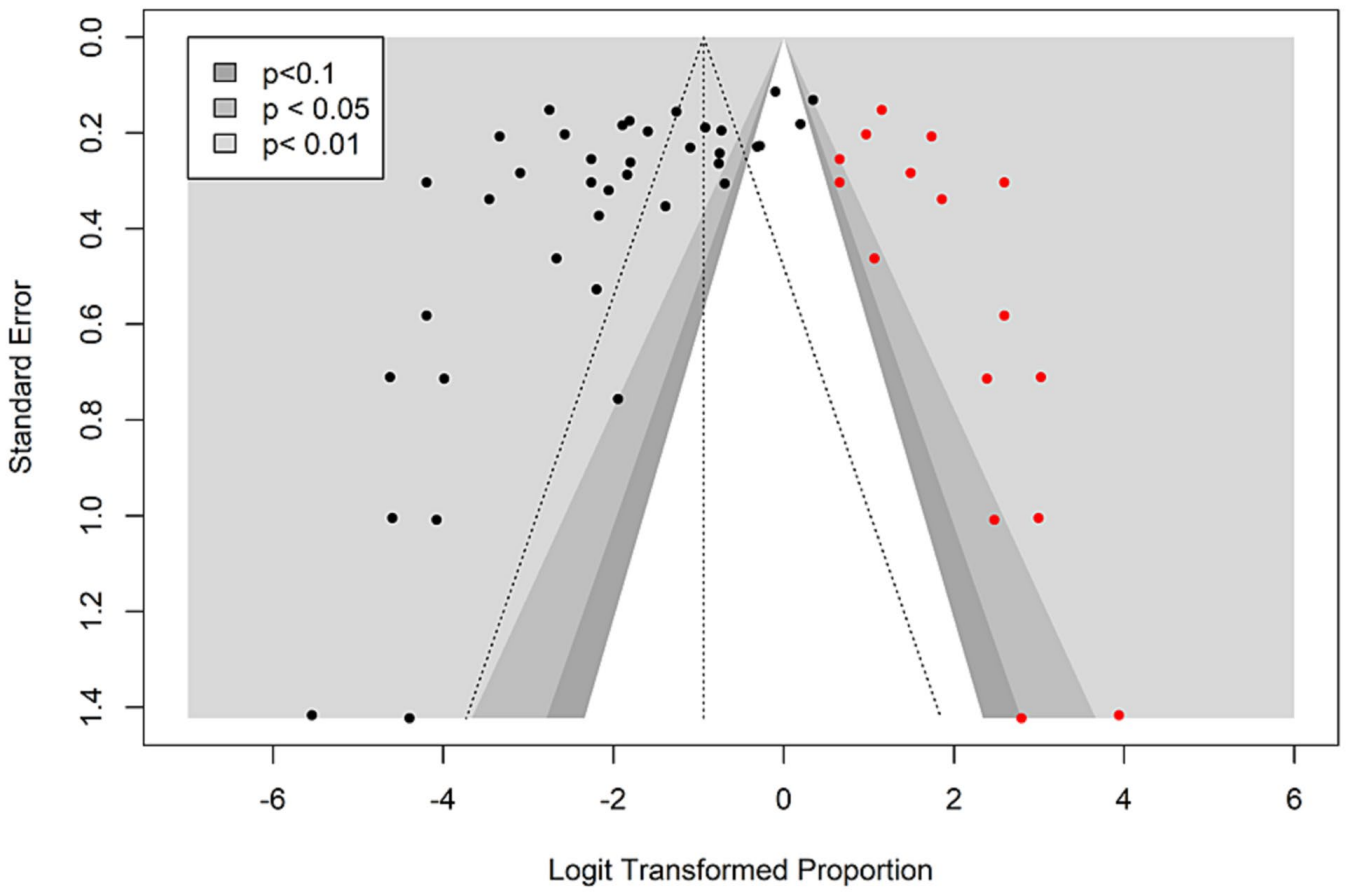
Contour-enhanced funnel plot indicating the publication bias for the prevalence studies on seafood-borne *Vibrio vulnificus* in the Asia countries. Black dots represent the studies included in this meta-analysis and red dots represent the missing studies added by the trim- and-fill method.

To investigate the extent of publication bias for the prevalence of seafood-borne *V. vulnificus* in Asia, a contour-enhanced funnel plot with different significance levels (<0.1, <0.05, <0.01) was generated with the logit-transformed proportion on the *x*-axis and their corresponding standard errors on the *y*-axis. A visual inspection of the funnel plot revealed an asymmetric distribution of the studies on both sides of the mean effect, suggesting the likelihood of publication bias (Figure 6). The presence of funnel plot asymmetry was further confirmed by Egger’s regression test that yielded a statistically significant *p*-value (*t* = −3.483, *p* = 0.001), suggesting the presence of publication bias for the prevalence of *V. vulnificus* in seafood in Asia. Applying the trim-and-fill method, 16 missing studies were identified, leading to a notable shift in the pooled prevalence estimate from 10.47% (CI: 6.8–15.8) to 28.07% (CI: 17.89–41.15). However, the shift in the prevalence estimate did not result from the inclusion of the original data, so it would not affect the validity of these findings. It rather underscores the importance of conducting additional studies to enhance the knowledge of the current prevalence status of *V. vulnificus* in seafood.

## 4 Discussion

*Vibrio vulnificus* is a highly lethal natural contaminant in marine ecosystems, particularly in warm climate zones, with an optimum growth temperature of 25°C (Chase and Harwood, 2011; Oliver, 2015). It is the leading cause of seafood-related deaths in the USA, with an annual estimate of 93 outbreaks and 36 deaths (Panicker et al., 2004; Scallan et al., 2011). This elevated incidence rate probably results from the consumption of raw seafood or a higher prevalence of predisposing factors (Drake et al., 2007). Many Asian countries have also reported multiple cases of *V. vulnificus* infection (World Health Organization, 2020). The Korean Center for Disease Control and Prevention estimates 40–70 confirmed cases annually in Korea (Drake et al., 2007). Inoue et al. (2008) estimated 12–24 cases per year in Japan and Taiwan had a peak occurrence of 26 cases per million population in 2000 (Hsueh et al., 2004). Furthermore, a few cases of *V. vulnificus* septicemia have been reported in Thailand (Thamlikitkul, 1990) and India (Saraswathi et al., 1989). These outbreaks are expected to increase in the future owing to the increasing trend in raw seafood consumption, a growing number of immunocompromised individuals, and the impact of anthropogenic activities and global warming on marine habitats (Forman et al., 2008; Baker-Austin et al., 2010). Owing to the morbidity and mortality caused by *V. vulnificus* in Asian countries (Oliver, 2015), this study was conducted to assess the prevalence of this pathogen in the regions with the most consumed seafood in Asia and to investigate the potential risk factors associated with its occurrence using a systematic review with meta-analysis and meta-regression approaches. The validity of studies included in the meta-analysis was evaluated by assessing the RoB against seven quality criteria established by the JBI for prevalence studies (Figures 1B,C). The findings indicated a 10.47% (980/7231, 95% CI: 6.8–15.8%) prevalence of seafood-borne *V. vulnificus* in Asia, which correlates with the prevalence reported in New Zealand (32/311, 10.3%) (Cruz et al., 2016). A lower prevalence rate of *V. vulnificus* has been reported in some European countries, including Spain (0/101, 0%) (Garrido-Maestu et al., 2016), Denmark (3/46, 7%) (Dalsgaard and Høi, 1997), Germany (1/160, 0.6%) (Vu et al., 2018), and African countries, such as Nigeria (2/150, 1.3%) (Adebayo-Tayo et al., 2011) and South Africa (0/85, 0%) (Kalule et al., 2019). This difference might result from variations in geographical location and climatic conditions across the Asian, European, and African regions. Another possible reason for the differences in prevalence rates could be disparities in sample size and study design. Furthermore, the varying levels of hygiene standards for seafood management in different countries might also be a cause of variation in the prevalence rate of *V. vulnificus* in seafood. Given the pathogenicity of *V. vulnificus* reported in several continents, the potential risk factors influencing its prevalence can be aggregated from the studies that address the same research question to establish efficient preventive measures (Baker-Austin et al., 2010).

In the subgroup analyses, the variation in countries of the included studies emerged as one of the prominent factors contributing to the disparities observed in the prevalence estimates across the studies. The studies conducted in East Asian countries such as Japan and China showed the highest prevalence rate (47.6 and 36.9% respectively). South Asian (India) and Southeast Asian (Thailand and Malaysia) countries had the second-highest prevalence rate (10–20%), whereas the lowest prevalence rate (<10%) was reported in West Asian countries such as Turkey and Iran (Figure 2). This difference might be associated with the variations in water temperature of the various coastal regions of Asia. Previous research has also reported that the major outbreaks of *V. vulnificus* infection in Asian countries, such as Japan, China, Korea, India, Thailand, and Malaysia, are attributed to their proximity to warmer marine environments, which raises the risk of such infections in these regions compared to that in the West Asian region (Saraswathi et al., 1989; Thamlikitkul, 1990; Song et al., 2020). In addition to the country, the detection method also revealed as a significant variable in moderator analyses. Univariate meta-regression showed its marked impact on the heterogeneity among the studies (*R*^2^ = 12.93%, *p* = 0.03), and the estimates of predictor importance also highlighted its substantial effect (*r* = 0.95) on the multivariate meta-regression model (Figure 4). Moreover, the subgroup analysis revealed a statistically significant variation (*p* = 0.03) in the prevalence rates between the studies using biochemical tests and those using PCR. This distinction can be attributed to the differential ability of biochemical tests and PCR assays to identify causative pathogens (Talaiekhozani, 2013). For instance, biochemical tests such as enzymatic detection and carbohydrate fermentation tests are commonly used to confirm the presence of *V. vulnificus* based on its characteristic metabolic activities (Loo et al., 2022). Although these tests are considered standard methods for the species identification of the pathogens, the phenotypic plasticity exhibited by *V. vulnificus* complicates the biochemical identification procedures, which can lead to misidentifications and false negative or false positive results in the characterization of microbial infections (Law et al., 2015). Conversely, PCR methods, with their ability to target specific genes such as *V. vulnificus* hemolysin alpha gene (*vvhA*), offer enhanced accuracy of results (Harwood et al., 2004). The disparities in equipment quality, PCR devices, reagent purity, laboratory protocols, and sample pretreatment methods can also introduce inconsistencies in reported prevalence rates across the studies (Miller et al., 2011). Thus caution should be exercised in selecting the appropriate detection method as these methodological variations can significantly influence the prevalence estimates. Owing to their high sensitivity, specificity, time-efficiency, and reduced labor intensity, the researchers also prefer rapid and simultaneous PCR methods over the more time-consuming and labor-intensive biochemical tests for identifying microbes (Loo et al., 2022).

For the sample size, the subgroup analysis indicated that the prevalence studies with small sample numbers reported a higher prevalence rate (12.63, 95%CI: 7.34–20.87) than the studies with large sample numbers (8.05, 95%CI: 4.11–15.18). This difference could be related to the fact that due to statistical uncertainty, the studies with small sample sizes tend to have higher effect sizes than those detected in the large studies (Slavin and Smith, 2009). In addition, univariate meta-regression analysis revealed that sample size exerted a 9.5% influence on heterogeneity among the studies (*R*^2^ = 9.5%, *p* = 0.1) and its notable impact was also observed on the multivariate meta-regression model (*r* = 0.45). As a result, it was speculated that variations in the sample size could potentially influence the observed differences in the prevalence rate of *V. vulnificus* across the studies. Similarly, for the sampling stage, the subgroup analysis revealed that the prevalence of *V. vulnificus* in freshly harvested samples was 7.26% (95%CI: 3.5–14.47%), while a notably higher prevalence rate of 13.49% (95%CI: 8.2–21.41%) was observed in the samples collected at the post-harvest stage. This observed rise in prevalence can be attributed to the continued growth of *V. vulnificus* in post-harvested samples during transport. DaSilva et al. (2012) also reported that the prolonged interval between harvesting and transportation to the seafood market, occurring over a temperature range of 15 to 30°C, facilitates the growth of *V. vulnificus* from 0.024 to 0.103 log MPN/h in contaminated seafood samples. This escalation of *V. vulnificus* concentration is likely to result in reaching or surpassing the detection limit, thereby contributing to the heightened prevalence rate observed in samples collected during the post-harvest stage. In the data visualization graphs, the scatter plot displays the simultaneous influence of publication year, detection method, and sampling stage, offering a comprehensive insight into the combined effects of these variables on the prevalence rate. It was observed that the studies reported a lower prevalence rate after 2015 irrespective of the variations in sampling stage and detection method (Figure 5A). The subgroup analysis based on the publication year also showed a significant decline in the prevalence rate of *V. vulnificus* after 2015 (Supplementary Table S5). This may indicate improved management practices in the handling, packaging, processing, and distribution of seafood after 2015, which likely contributed to the reduction in prevalence rate, as previously reported (Chan et al., 1989; Nie et al., 2022). However, that prevalence rates were still influenced by other variables such as sample type and sample size. Regarding sample type, it was observed that 41% of samples analyzed after 2015 were fish, which tend to exhibit lower prevalence rates than shellfish (Potasman et al., 2002; Adebayo-Tayo et al., 2011). In the case of sample size, 50% of studies conducted after 2015 utilized large sample sizes (>150), which are typically associated with lower prevalence rates (Slavin and Smith, 2009). In conclusion, the results suggest that, despite the emerging threat of *V. vulnificus* prevalence in Asia, the low prevalence rates observed after 2015 in this study reflect a complex interplay among various factors, such as high sample size, less contaminated fish samples, and proper seafood management. However, *V. vulnificus* remains a prevalent pathogen in Asia, underscoring the continued need for further research and vigilance to promote seafood safety. Figure 5B depicts the prevalence of *V. vulnificus* across various sample types included in this meta-analysis. It was observed that the majority of shellfish samples, including oysters, mussels, and clams, exhibited a higher prevalence of *V. vulnificus* than fish samples. This high prevalence can be attributed to the loose textured flesh and filter feeding practice of shellfish, which results in the higher accumulation of *V. vulnificus* in their bodies as compared to fish (Potasman et al., 2002).

Implementing various preventive measures throughout the seafood supply chain can effectively reduce the contamination of *V. vulnificus*, thereby mitigating the risk of infections associated with seafood consumption. The level of *V. vulnificus* contamination in seafood samples can increase or decrease from harvest to consumption pathway, depending upon the environmental conditions, such as storage temperature, storage time, and depuration methods. For instance, the concentration of *V. vulnificus* can be below the limit of detection (<110 CFU/g) in positive seafood samples at the pre-harvest stage, while the exposure to temperatures between 15 and 30°C at the post-harvest stage can result in level reaching to 2.5 × 10^5^ CFU/g due to the continued growth of *V. vulnificus* (Birkenhauer and Oliver, 2003). It is essential to store the seafood samples in iced containers, maintained at a temperature below 13°C after harvesting, instead of leaving them on the boat deck to reduce the growth of *V. vulnificus* and prevent any biological, chemical, or physical damage (Ryder et al., 2014). In addition, the seafood samples should be washed with clean water under high hydrostatic pressure before storage to inactivate the pathogens harboring in them (Cook, 2003). As the concentration of *V. vulnificus* in seafood is also expected to increase during transportation to the retail market, maintaining the storage temperature (~13°C) during transportation is necessary to prevent the proliferation of *V. vulnificus* (FAO/WHO, 2005). Upon reaching the market, proper refrigeration of seafood is vital to reduce the growth of *V. vulnificus*; however, the acclimatization of *V. vulnificus* even at 15°C could diminish the potency of refrigeration. Thus, a combination of vacuum packaging and freezing is recommended to enhance the efficacy of preservation (Parker et al., 1994; Bryan et al., 1999). Freezing is often adopted for long-term storage since the prolonged refrigeration of seafood at −40°C for 3 weeks could lead to a substantial reduction in the *V. vulnificus* number (Ryder et al., 2014). At the consumption level, thermal treatment would be required for minimizing the contamination considering that the standard methods for depuration of seafood such as washing and scrubbing have not been effective in reducing the level of the pathogens in raw or slightly processed seafood (Froelich and Noble, 2014). Cook and Ruple (1992) demonstrated that *V. vulnificus* can be reduced to non-detectable levels in shucked shellfish by exposing them to 50°C for 10 min. In addition to temperature, salinity is a critical factor in controlling the level of *V. vulnificus*. Submerging seafood in strong briny water for a few hours before cooking can significantly decrease the concentration of *V. vulnificus* owing to its inability to survive in high-salinity environments (Sampels, 2015). In summary, reducing the risk of *V. vulnificus* infection from seafood consumption requires proper handling practices across the entire seafood supply chain, coupled with careful consumption habits, thereby safeguarding the health and well-being of consumers.

Generally, the studies with positive and significant (*p* < 0.05) results are more likely to be published compared to the studies with small sample sizes or negative effect sizes, resulting in a publication bias. The publication bias in this meta-analysis (Figure 6) probably resulted from the fact that the included studies were all collected through electronic database searches; hence, the possibility of missing some studies cannot be ruled out. The tools designed to assess publication bias have been developed for randomized controlled trials (RCTs) in which the publication is affected by statistical significance levels (Maulik et al., 2011). While the current meta-analysis estimating the prevalence of *V. vulnificus*, only observational (e.g., cross-sectional) and non-comparative studies were included, and thus significance levels were not necessary. Since the available tools to investigate publication bias are generally considered less useful in the context of meta-analyses of prevalence studies, the detected publication bias in this study does not necessarily undermine the validity of the findings (Wang, 2017).

Although meta-analysis is a useful tool for evaluating the pooled prevalence, this study has several limitations. First, other relevant covariates, such as water temperature, salinity, water quality, harvesting/sampling season, and farming conditions, which can influence the modeling process and explain the between-study variation (Parlapani, 2021), were not sufficiently reported in the selected primary studies, despite the quality of their work. Thus, because of insufficient details on the environmental characteristics of the sampling locations reported in the primary studies, the results should be interpreted with caution. Second, only 38 studies including 7,231 samples were eligible for meta-analysis, which may be considered an insufficient number to draw conclusions for the entire Asian continent. In addition, the variability in the prevalence rate at some stages of the seafood supply chain, such as transportation, storage, and consumption, was not considered in this study due to limited data availability. Future studies incorporating comprehensive environmental data at each stage of the supply chain are warranted to improve the understanding of *V. vulnificus* prevalence dynamics in Asian seafood. Finally, the threat of *V. vulnificus* infections associated with seafood consumption cannot be entirely viewed in terms of the prevalence rates. Several other aspects including the pathogen concentration, seafood handling practices, consumption frequency, consumption behavior, and dose–response relationship must be considered in future risk assessment studies of *V. vulnificus* infections. Despite these limitations, the present study give evidence-based information on the prevalence of *V. vulnificus* in seafood and its associated risk factors in Asia, which can provide crucial baseline data for future risk assessment of *V. vulnificus* infections.

In conclusion, despite the heterogeneous prevalence rates among the studies included in this meta-analysis, the overall prevalence estimate, coupled with the results from subgroup and regression analyses offers a comprehensive understanding of the current prevalence and risk factors of *V. vulnificus* in seafood across Asia, based on the available literature data. The findings also highlight the need for improved hygiene practices during seafood handling to reduce the risk of contamination and ensure public safety. Continued and coordinated efforts by various agencies, including those involved in water quality, disease surveillance, consumer education, and seafood management, are required to reduce seafood-borne disease outbreaks. Global implementation of Hazard Analysis and Critical Control Point (HACCP) systems is imperative for the complete food chain, from water to tables. Another essential approach involves the regulation of the use of antibiotics in aquaculture to prevent the development of antimicrobial-resistant strains of *V. vulnificus*. Further cross-sectional studies with large sample sizes are necessary to enhance the information on the incidence, prevalence, and risk factors of *V. vulnificus* in seafood.

## Data availability statement

The original contributions presented in the study are included in the article/Supplementary material, further inquiries can be directed to the corresponding author.

## Author contributions

MT: Data curation, Formal analysis, Investigation, Methodology, Software, Validation, Visualization, Writing – original draft, Writing – review & editing. EN: Data curation, Formal analysis, Investigation, Resources, Writing – review & editing. GW: Conceptualization, Funding acquisition, Investigation, Project administration, Supervision, Writing – review & editing.

## Footnotes

^1^http://www.fric.kr/user/centerMainView.do?centerId=jbnu

## References

[ref1] Adebayo-Tayo B. Okonko I. Esen C. Odu N. Onoh C. Igwiloh N. (2011). Incidence of potentially pathogenic Vibrio spp. in fresh seafood from Itu Creek in Uyo, Akwa Ibom state, Nigeria. World Appl. Sci. J. 15, 985–991.

[ref2] Amalina N. Z. Dzarifah Z. Amal M. N. A. Yusof M. T. Zamri-Saad M. Al-saari N. . (2019). Recent update on the prevalence of Vibrio species among cultured grouper in peninsular Malaysia. Aquac. Res. 50, 3202–3210. doi: 10.1111/are.14275

[ref3] Baker-Austin C. Stockley L. Rangdale R. Martinez-Urtaza J. (2010). Environmental occurrence and clinical impact of Vibrio vulnificus and Vibrio parahaemolyticus: a European perspective. Environ. Microbiol. Rep. 2, 7–18. doi: 10.1111/j.1758-2229.2009.00096.x, PMID: 23765993

[ref4] Barendregt J. J. Doi S. A. Lee Y. Y. Norman R. E. Vos T. (2013). Meta-analysis of prevalence. J. Epidemiol. Community Health 67, 974–978. doi: 10.1136/jech-2013-203104 23963506

[ref5] Birkenhauer J. M. Oliver J. D. (2003). Use of diacetyl to reduce the load of Vibrio vulnificus in the eastern oyster, Crassostrea virginica. J. Food Prot. 66, 38–43. doi: 10.4315/0362-028X-66.1.38, PMID: 12540179

[ref6] Bross M. H. Soch K. Morales R. Mitchell R. B. (2007). Vibrio vulnificus infection: diagnosis and treatment. Am. Fam. Physician 76, 539–544. PMID: 17853628

[ref7] Bryan P. J. Steffan R. J. DePaola A. Foster J. W. Bej A. K. (1999). Adaptive response to cold temperatures in Vibrio vulnificus. Curr. Microbiol. 38, 168–175. doi: 10.1007/PL00006782, PMID: 9922468

[ref8] Chan K. Y. Woo M. L. Lam L. Y. French G. L. (1989). Vibrio parahaemolyticus and other halophilic vibrios associated with seafood in Hong Kong. J. Appl. Bacteriol. 66, 57–64. doi: 10.1111/j.1365-2672.1989.tb02454.x, PMID: 2722715

[ref9] Chase E. Harwood V. J. (2011). Comparison of the effects of environmental parameters on growth rates of Vibrio vulnificus biotypes I, II, and III by culture and quantitative PCR analysis. Appl. Environ. Microbiol. 77, 4200–4207. doi: 10.1128/AEM.00135-11, PMID: 21515718 PMC3131657

[ref10] Chung P. Chuang S. Tsang T. Wai-Man L. Yung R. Lo J. . (2006). Cutaneous injury and Vibrio vulnificus infection. Emerg. Infect. Dis. 12, 1302–1303. doi: 10.3201/eid1208.051495, PMID: 16972360 PMC3291212

[ref11] Coerdt K. M. Khachemoune A. (2021). Vibrio vulnificus: review of mild to life-threatening skin infections. Cutis 107, E12–E17. doi: 10.12788/cutis.0183, PMID: 33891847

[ref12] Cook D. W. (2003). Sensitivity of Vibrio species in phosphate-buffered saline and in oysters to high-pressure processing. J. Food Prot. 66, 2276–2282. doi: 10.4315/0362-028X-66.12.2276, PMID: 14672224

[ref13] Cook D. W. Ruple A. D. (1992). Cold storage and mild heat treatment as processing aids to reduce the numbers of Vibrio vulnificus in raw oysters. J. Food Prot. 55, 985–989. doi: 10.4315/0362-028X-55.12.985, PMID: 31084103

[ref14] Crowther M. Lim W. Crowther M. A. (2010). Systematic review and meta-analysis methodology. J. Am. Soc. Hematol. 116, 3140–3146. doi: 10.1182/blood-2010-05-280883 20656933

[ref15] Cruz C. Chycka M. Hedderley D. Fletcher G. (2016). Prevalence, characteristics and ecology of Vibrio vulnificus found in New Zealand shellfish. J. Appl. Microbiol. 120, 1100–1107. doi: 10.1111/jam.13064, PMID: 26788798

[ref16] Dalsgaard A. Høi L. (1997). Prevalence and characterization of Vibrio vulnificus isolated from shrimp products imported into Denmark. J. Food Prot. 60, 1132–1134. doi: 10.4315/0362-028X-60.9.1132, PMID: 31207820

[ref17] DaSilva L. Parveen S. DePaola A. Bowers J. Brohawn K. Tamplin M. L. (2012). Development and validation of a predictive model for the growth of Vibrio vulnificus in postharvest shellstock oysters. Appl. Environ. Microbiol. 78, 1675–1681. doi: 10.1128/AEM.07304-11, PMID: 22247136 PMC3298140

[ref18] Deeks J. J. Higgins J. P. Altman D. G.., and Group C.S.M. (2019). Analysing data and undertaking meta-analyses. Cochrane handbook for systematic reviews of interventions, 241–284

[ref19] Drake S. L. DePaola A. Jaykus L. A. (2007). An overview of Vibrio vulnificus and Vibrio parahaemolyticus. Compr. Rev. Food Sci. Food Saf. 6, 120–144. doi: 10.1111/j.1541-4337.2007.00022.x

[ref20] Egger M. Smith G. D. Schneider M. Minder C. (1997). Bias in meta-analysis detected by a simple, graphical test. BMJ 315, 629–634. doi: 10.1136/bmj.315.7109.629, PMID: 9310563 PMC2127453

[ref21] FAO/WHO (2005). Risk assessment of Vibrio vulnificus in raw oysters: Interpretative summary and technical report. United Nation: World Health Organization.

[ref22] Field A. P. Gillett R. (2010). How to do a meta-analysis. Br. J. Math. Stat. Psychol. 63, 665–694. doi: 10.1348/000711010X502733 20497626

[ref23] Forman S. Hungerford N. Yamakawa M. Yanase T. Tsai H. Joo Y. . (2008). Climate change impacts and risks for animal health in Asia. Rev Sci Tech Off Int Epiz 27, 581–597. doi: 10.20506/rst.27.2.1814, PMID: 18819679

[ref24] Froelich B. A. Noble R. T. (2014). Factors affecting the uptake and retention of Vibrio vulnificus in oysters. Appl. Environ. Microbiol. 80, 7454–7459. doi: 10.1128/AEM.02042-14, PMID: 25261513 PMC4249221

[ref25] Garrido-Maestu A. Lozano-León A. Rodríguez-Souto R. R. Vieites-Maneiro R. Chapela M.-J. Cabado A. G. (2016). Presence of pathogenic Vibrio species in fresh mussels harvested in the southern rias of Galicia (NW Spain). Food Control 59, 759–765. doi: 10.1016/j.foodcont.2015.06.054

[ref26] Harrer M. Cuijpers P. Furukawa T. A. Ebert D. D. (2021). Doing meta-analysis with R: a hands-on guide Chapman and Hall/CRC.

[ref27] Harwood V. J. Gandhi J. P. Wright A. C. (2004). Methods for isolation and confirmation of Vibrio vulnificus from oysters and environmental sources: a review. J. Microbiol. Methods 59, 301–316. doi: 10.1016/j.mimet.2004.08.001, PMID: 15488274

[ref28] Heng S.-P. Letchumanan V. Deng C.-Y. Ab Mutalib N.-S. Khan T. M. Chuah L.-H. . (2017). Vibrio vulnificus: an environmental and clinical burden. Front. Microbiol. 8:997. doi: 10.3389/fmicb.2017.00997, PMID: 28620366 PMC5449762

[ref29] Higgins J. P. Thompson S. G. (2002). Quantifying heterogeneity in a meta-analysis. Stat. Med. 21, 1539–1558. doi: 10.1002/sim.1186, PMID: 12111919

[ref30] Hsueh P.-R. Lin C.-Y. Tang H.-J. Lee H.-C. Liu J.-W. Liu Y.-C. . (2004). Vibrio vulnificus in Taiwan. EID 10, 1363–1368. doi: 10.3201/eid1008.040047 PMC3320410 15496235

[ref31] Inoue Y. Ono T. Matsui T. Miyasaka J. Kinoshita Y. Ihn H. (2008). Epidemiological survey of Vibrio vulnificus infection in Japan between 1999 and 2003. J. Dermatol. 35, 129–139. doi: 10.1111/j.1346-8138.2008.00432.x, PMID: 18346255

[ref32] Jones M. K. Oliver J. D. (2009). Vibrio vulnificus: disease and pathogenesis. Infect. Immun. 77, 1723–1733. doi: 10.1128/IAI.01046-08, PMID: 19255188 PMC2681776

[ref33] Kalule J. B. Smith A. M. Vulindhlu M. Tau N. P. Nicol M. P. Keddy K. H. . (2019). Prevalence and antibiotic susceptibility patterns of enteric bacterial pathogens in human and non-human sources in an urban informal settlement in Cape Town, South Africa. BMC Microbiol. 19, 1–11. doi: 10.1186/s12866-019-1620-6 31694551 PMC6836408

[ref34] Law J. W.-F. Ab Mutalib N.-S. Chan K.-G. Lee L.-H. (2015). Rapid methods for the detection of foodborne bacterial pathogens: principles, applications, advantages and limitations 5, 5. doi: 10.3389/fmicb.2014.00770, PMID: 25628612 PMC4290631

[ref35] Lin L. Chu H. (2020). Meta-analysis of proportions using generalized linear mixed models. Epidemiology 31:713. 32657954 10.1097/EDE.0000000000001232 PMC7398826

[ref36] Lipsey M. W. Wilson D. B. (2001). Practical meta-analysis Thousand Oaks, CA: SAGE publications, Inc.

[ref37] Liu J.-W. Lee K. Tang H.-J. Ko W.-C. Lee H.-C. Liu Y.-C. . (2006). Prognostic factors and antibiotics in Vibrio vulnificus septicemia. Arch. Intern. Med. 166, 2117–2123. doi: 10.1001/archinte.166.19.2117, PMID: 17060542

[ref38] Loo K.-Y. Law J. W.-F. Tan L. T.-H. Pusparajah P. Letchumanan V. Lee L.-H. (2022). Diagnostic techniques for rapid detection of Vibrio species. Aquaculture 561:738628. doi: 10.1016/j.aquaculture.2022.738628

[ref39] Lydon K. A. Kinsey T. Le C. Gulig P. A. Jones J. L. (2021). Biochemical and virulence characterization of Vibrio vulnificus isolates from clinical and environmental sources. Front. Cell. Infect. Microbiol. 11:637019. doi: 10.3389/fcimb.2021.637019, PMID: 33718284 PMC7952748

[ref40] Martin J.. (2017). © Joanna Briggs Institute 2017 Critical Appraisal Checklist for Prevalence Studies. Published online 7.

[ref41] Maulik P. K. Mascarenhas M. N. Mathers C. D. Dua T. Saxena S. (2011). Prevalence of intellectual disability: a meta-analysis of population-based studies. Res. Dev. Disabil. 32, 419–436. doi: 10.1016/j.ridd.2010.12.018, PMID: 21236634

[ref42] Miller W. G. Jones G. R. Horowitz G. L. Weykamp C. (2011). Proficiency testing/external quality assessment: current challenges and future directions. Clin. Chem. 57, 1670–1680. doi: 10.1373/clinchem.2011.168641, PMID: 21965556

[ref43] Miyamoto K. Kawano H. Okai N. Hiromoto T. Miyano N. Tomoo K. (2021). Iron-utilization system in Vibrio vulnificus M2799. Mar. Drugs 19:710. doi: 10.3390/md19120710, PMID: 34940709 PMC8706444

[ref44] Naing L. Winn T. Rusli B. (2006). Practical issues in calculating the sample size for prevalence studies. Arch. Orofac. Sci. 1, 9–14.

[ref45] Nie X. Zhang R. Cheng L. Zhu W. Li S. Chen X. (2022). Mechanisms underlying the deterioration of fish quality after harvest and methods of preservation. Food Control 135:108805. doi: 10.1016/j.foodcont.2021.108805

[ref46] Oliver J. D. (2015). The biology of Vibrio vulnificus. Microbiol. Spectr. 3:e3. doi: 10.1128/microbiolspec.VE-0001-2014 26185084

[ref48] Page M. J. Moher D. Bossuyt P. M. Boutron I. Hoffmann T. C. Mulrow C. D. . (2021). PRISMA 2020 explanation and elaboration: updated guidance and exemplars for reporting systematic reviews. BMJ 372. 160. 10.1136/bmj.n160 PMC8005925 33781993

[ref49] Palmer T. M. Sutton A. J. Peters J. L. Moreno S. G. (2008). Contour-enhanced funnel plots for meta-analysis. Stata J. 8, 242–254. doi: 10.1177/1536867X0800800206

[ref50] Panicker G. Myers M. L. Bej A. K. (2004). Rapid detection of Vibrio vulnificus in shellfish and Gulf of Mexico water by real-time PCR. Appl. Environ. Microbiol. 70, 498–507. doi: 10.1128/AEM.70.1.498-507.2004, PMID: 14711681 PMC342803

[ref51] Parker R. W. Maurer E. M. Childers A. B. Lewisi D. H. (1994). Effect of frozen storage and vacuum-packaging on survival of Vibrio vulnificus in Gulf Coast oysters (Crassostrea virginica). J. Food Prot. 57, 604–606. doi: 10.4315/0362-028X-57.7.604, PMID: 31121704

[ref52] Parlapani F. F. (2021). Microbial diversity of seafood. Curr. Opin. Food Sci. 37, 45–51. doi: 10.1016/j.cofs.2020.09.005

[ref53] Potasman I. Paz A. Odeh M. (2002). Infectious outbreaks associated with bivalve shellfish consumption: a worldwide perspective. Clin. Infect. Dis. 35, 921–928. doi: 10.1086/342330, PMID: 12355378

[ref54] Ryder J. Iddya K. Ababouch L. (2014). Assessment and management of seafood safety and quality: current practices and emerging issues. FAO fisheries and aquaculture technical paper 574

[ref55] Sampels S. (2015). The effects of processing technologies and preparation on the final quality of fish products. Trends Food Sci. Technol. 44, 131–146. doi: 10.1016/j.tifs.2015.04.003

[ref56] Saraswathi K. Barve S. Deodhar L. (1989). Septicaemia due to Vibrio vulnificus. Trans. R. Soc. Trop. Med. Hyg. 83:714. doi: 10.1016/0035-9203(89)90406-9 2617638

[ref57] Scallan E. Hoekstra R. M. Angulo F. J. Tauxe R. V. Widdowson M.-A. Roy S. L. . (2011). Foodborne illness acquired in the United States—major pathogens. Emerg. Infect. Dis. 17, 7–15. doi: 10.3201/eid1701.P11101, PMID: 21192848 PMC3375761

[ref58] Slavin R. Smith D. (2009). The relationship between sample sizes and effect sizes in systematic reviews in education. Educ. Eval. Policy Anal. 31, 500–506. doi: 10.3102/0162373709352369

[ref59] Song X. Zang J. Yu W. Shi X. Wu Y. (2020). Occurrence and identification of pathogenic Vibrio contaminants in common seafood available in a Chinese traditional market in Qingdao. Front. Microbiol. 11:1488. doi: 10.3389/fmicb.2020.01488, PMID: 32695094 PMC7338590

[ref60] Strom M. S. Paranjpye R. N. (2000). Epidemiology and pathogenesis of Vibrio vulnificus. Microbes Infect. 2, 177–188. doi: 10.1016/S1286-4579(00)00270-7 10742690

[ref61] Talaiekhozani A. (2013). Guidelines for quick application of biochemical tests to identify unknown bacteria. Acc. Biotechnol. Res. 2013:e35. doi: 10.2139/ssrn.4101035

[ref62] Thamlikitkul V. (1990). Vibrio bacteremia in Siriraj Hospital. J. Med. Assoc. Thailand 73, 136–139. PMID: 2380645

[ref63] Thompson S. G. Higgins J. P. (2002). How should meta-regression analyses be undertaken and interpreted? Stat. Med. 21, 1559–1573. doi: 10.1002/sim.1187 12111920

[ref64] Vu T. T. T. Alter T. Huehn S. (2018). Prevalence of Vibrio spp. in retail seafood in Berlin, Germany. J. Food Prot. 81, 593–597. doi: 10.4315/0362-028X.JFP-17-366, PMID: 29517352

[ref65] Wai C. Y. Leung N. Y. Leung A. S. Wong G. W. Leung T. F. (2021). Seafood allergy in Asia: geographical specificity and beyond. Front. Allergy 2:903. doi: 10.3389/falgy.2021.676903 PMC8974776 35387013

[ref66] Wang N. (2017). Conducting meta-analyses of proportions in R. Research Gate: College Station, TX, USA

[ref67] Wang N. (2023). Conducting meta-analyses of proportions in R. J. Behav. Data Sci. 3, 64–126. doi: 10.35566/jbds/v3n2/wang

[ref47] World Health Organization (2020). Risk assessment tools for Vibrio parahaemolyticus and Vibrio vulnificus associated with seafood

[ref68] Zangoei-Fard S. Rahimi E. Shakerian A. (2020). Incidence and phenotypic pattern of antibiotic resistance of Vibrio species isolated from seafood samples caught from the Persian Gulf. Egypt. J. Vet. Sci. 51, 337–347. doi: 10.21608/ejvs.2020.26304.1164

