# Supplementary material for: Prevalence and risk factors of seafood-borne Vibrio vulnificus in Asia: a systematic review with meta-analysis and meta-regression
Source: Front Microbiol. 2024 Mar 6;15:1363560. doi: 10.3389/fmicb.2024.1363560 (PMC10951106; doi:10.3389/fmicb.2024.1363560)

Supplementary Figure 1. Sensitivity analysis of the prevalence of seafood-borne *Vibrio vulnificus* in Asia. The prevalence estimates (right) and their corresponding 95% confidence intervals were computed by leaving out each study (left).

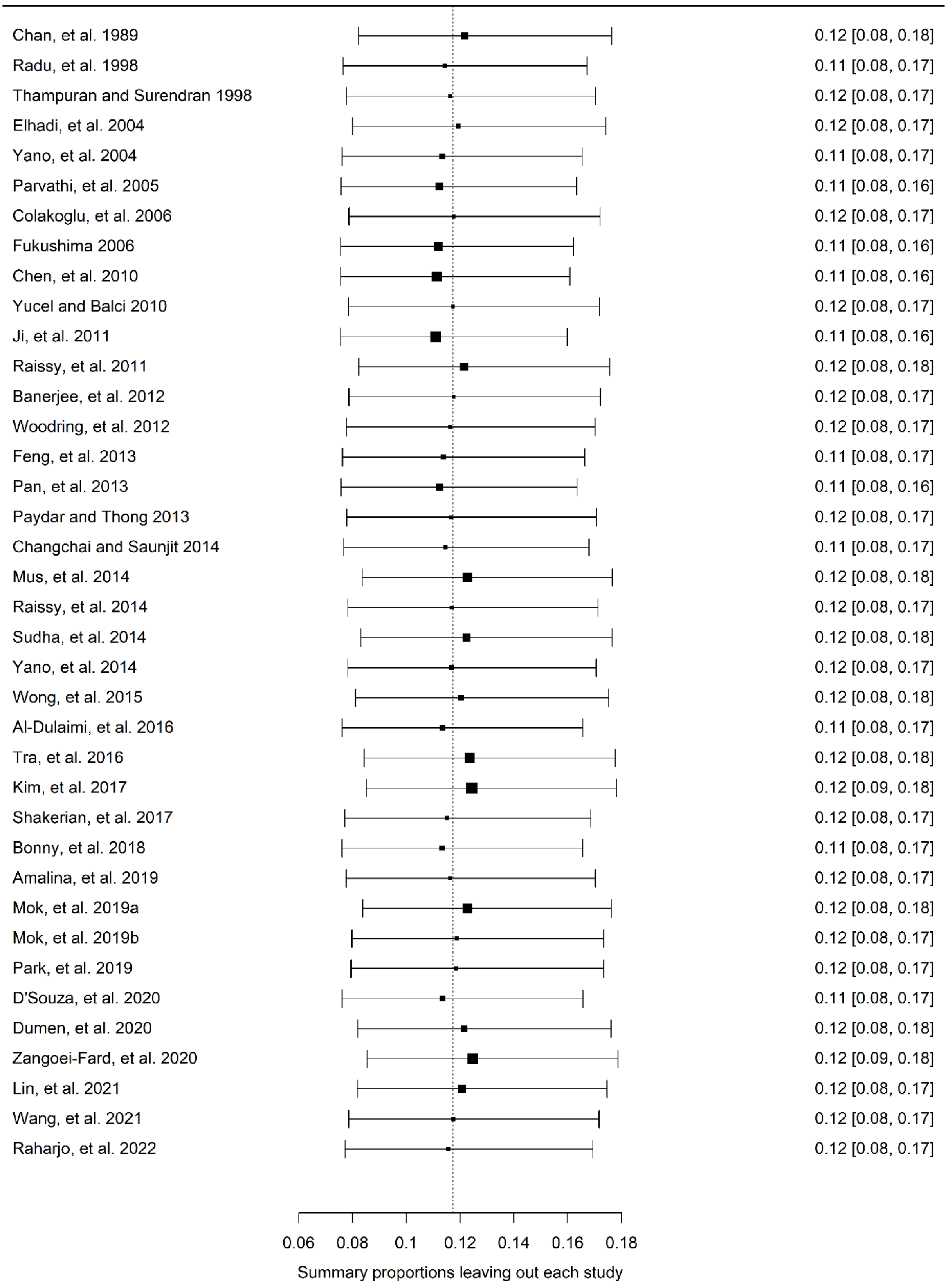

Supplement: Supplementary file 6 [file Data_Sheet_1.PDF]
